# Crystal structure of 2-(aza­niumylmeth­yl)pyridinium bis(hydrogen squarate)

**DOI:** 10.1107/S2056989017004376

**Published:** 2017-03-24

**Authors:** Nina Salamzadeh, Zeynep Demircioglu, Ufuk Korkmaz, Orhan Büyükgüngör

**Affiliations:** a Department of Physics, Faculty of Arts and Sciences, Ondokuz Mayıs University, Kurupelit, Samsun 55139, Turkey

**Keywords:** crystal structure, 2-(aza­niumylmeth­yl)pyridinium, squaric acid, hydrogen bonding

## Abstract

The asymmetric unit of the title compound, C_6_H_10_N_2_^2+^·2C_4_HO_4_^−^, comprises two hydrogen squarate (Hsq^−^; systematic name: 2-hy­droxy-3,4-dioxo­cyclo­butano­late) anions and a 2-(aza­niumylmeth­yl)pyridinium dication. The squaric acid mol­ecules each donate an H atom to the N atoms of the pyridine ring and the amino­methyl units of a 2-(amino­meth­yl)pyridine mol­ecule, forming the 1:2 salt. The Hsq^−^ anions are linked by strong O—H⋯O hydrogen bonds and an N—H⋯O hydrogen bond links the 2-(aza­niumylmeth­yl)pyridinium cation to one of the squaric acid anions. The crystal structure features additional N—H⋯O and O—H⋯O hydrogen bonds, π–π stacking and unusual weak C—O⋯π(ring) inter­actions.

## Abstract 2

The structure of the title squarate salt is reported. Classical N—H⋯O and O—H⋯O hydrogen bonds combine with weak C—O⋯π(ring) and π–π contacts to stabilize the crystal packing.

## Chemical context   

Hydrogen bonding is the most common way of generating supra­molecular organic systems in crystal engineering and mol­ecular recognition. Hydrogen-bonded systems generated from organic cations and anions are of special inter­est as they would be expected to form stronger hydrogen bonds than those in neutral mol­ecules (Reetz *et al.*,1994 ▸; Bertolasi *et al.*, 2001 ▸). Squaric acid (H_2_C_4_O_4_, H_2_sq), has been of inter­est because of its cyclic structure and potential aromaticity and is used as a building block in crystal engineering due to the simplicity of the cyclic units. It can be found in three forms: uncharged H_2_sq, the Hsq^−^ monoanion and the sq^2−^ dianion. The mono- and dianions are often produced following deprotonation by amines (Lam & Mak, 2000 ▸; Mathew *et al.*, 2002 ▸). The squarate derivatives are almost flat because of the π-conjugation of their C—C and C—O bonds, and therefore their four oxygen atoms behave as planar (*sp* ^2^) electron donors of one or two lone pairs of electrons. Recently, we reported the synthesis and characterization of the same organo­ammonium squarate as the title compound but as a hydrate in the triclinic space group *P* 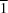 (Korkmaz & Bulut, 2013 ▸). We report here the unsolvated form of this salt, which crystallizes in the monoclinic space group *P*2_1_/*c*.
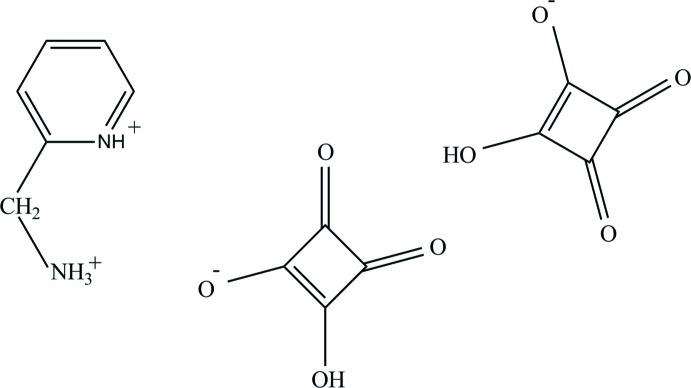


## Structural commentary   

**Figure 1:**
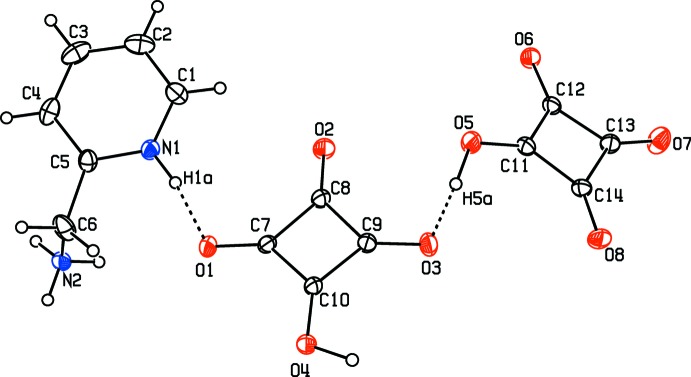
A view of the asymmetric unit of (I), showing the atom-numbering scheme and 30% probability displacement ellipsoids. Dashed lines indicate hydrogen bonds.

2-(Amino­meth­yl) pyridine forms a salt with two squaric acid mol­ecules and each mol­ecule of the acid loses one proton. One of these is transferred to the N atom of the pyridine ring, generating the 4-(amino­meth­yl)morpholinium mono-cation. The other from the second acid mol­ecule is engaged in the formation of a homo-conjugated hydrogen squarate anion *via* a short, symmetric O5—H5*A*⋯O3 [2.4583 (14) Å] hydrogen bond (Fig. 1 ▸). The electron density associated with this H atom is shared by the O5 and O3 atoms, indicating a large degree of ionic character (Gilli & Gilli, 2000 ▸). Considering the range (2.38–2.50 Å) of Gilli’s classification for such an inter­action, this hydrogen bonding can be referred to as negative charge-assisted hydrogen bonding [(−) CAHB] (Gilli & Gilli, 2009 ▸; Gilli *et al.*, 2001 ▸ *;* Becke, 1993 ▸, Lee *et al.*, 1988 ▸) and can be represented as [-O⋯H⋯O-]^−^.

N1/C1–C5, C7–C10 and C11–C14 are defined as rings 1, 2 and 3, respectively, with centroids *Cg*1, *Cg*2 and *Cg*3. The dihedral angles between the mean plane of ring 1 and those of rings 2 and 3 are 18.818 (8) and 31.564 (6)°, respectively. The dihedral angle between the two squarate anions is 29.19 (1)°. The angles between the C—C bonds in the Hsq^−^ anions are close to 90°, with the oxygen atoms directed almost along the diagonals.

The C—C distances in the planar squarate ring systems reflect partial double-bond character for C9—C10, C7—C10, C11—C12 and C11—C14 with distances of 1.4291 (17), 1.4357 (16), 1.4139 (17) and 1.4465 (18) Å, respectively. In contrast C7—C8, C8—C9, C12—C13 and C13—C14 display more single-bond character with distances of 1.4886 (17), 1.4929 (17), 1.4802 (18) and 1.5141 (17) Å, respectively. The Hsq^−^ ion has one C—O bond (C11—O5) at 1.3023 (17) Å, which is significantly longer than a normal single C—O bond. This most likely reflects the involvement in the negative charge-assisted hydrogen bonding mentioned earlier. At 1.3000 (15) Å, the C10—O4 bond is similarly extended. The remaining C—O bonds in both rings display a similar pattern with one obvious C=O double bond in each ring [C8=O2, 1.2268 (15) Å and C13=O7, 1.2141 (17) Å] and the others of inter­mediate length in the range 1.2356 (16) to 1.2658 (15) Å, indicating some delocalization occurring in both rings.

## Supra­molecular features   

**Figure 2:**
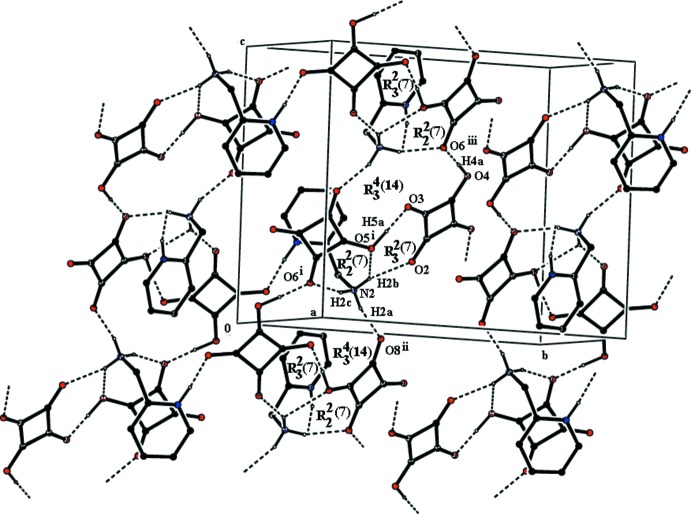
A view of the N—H⋯O and O—H⋯O inter­actions in the crystal of the title compound (hydrogen bonds are shown as dashed lines; see Table 1 ▸ for numerical details).

**Figure 3:**
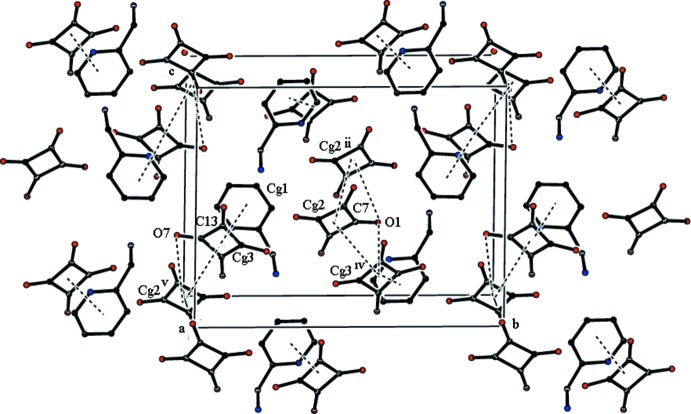
A packing diagram showing the C—O⋯π and π–π stacking inter­actions. [Symmetry codes: (ii) −*x* + 1, −*y* + 1, −*z* + 1; (iv) −*x* + 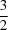, *y* + 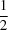, −*z* + 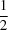; (v) −*x* + 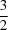, *y* − 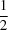, −*z* + 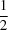.] H atoms not involved in the inter­actions have been omitted for clarity.

The two hydrogen squarate anions are linked in the asymmetric unit by a short hydrogen-bonding inter­action O5—H5*A*⋯O3 [2.4583 (14) Å] related to the proton-sharing inter­action discussed earlier. This pair of anions is further linked to the 2-(aza­niumylmeth­yl)pyridinium dication by an N1—H1*A*⋯O1 hydrogen bond, Fig. 1 ▸, Table 1 ▸. O5—H5*A*⋯O3, N2—H2*B*⋯O2^i^ and N2—H2*B*⋯O5^i^ hydrogen bonds form rings with an 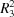
(7) graph-set motif while N2—H2*C*⋯O6^i^ and N2—H2*B*⋯O5^i^ hydrogen bonds combine to form 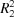
(7) rings. In addition, heteronuclear N2—H2*C*⋯O6^i^, and N2—H2*A*⋯O8^ii^ and homonuclear O4—H4*A*⋯O6^iii^ and O5—H5*A*⋯O3 hydrogen bonds generate a larger 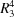
(14) ring motif (Fig. 2 ▸, Table 1 ▸). The crystal packing also features unusual weak C7—O1⋯*Cg*2^ii^, C7—O1⋯*Cg*3^iv^ and C13—O7⋯*Cg*2^v^ inter­actions reinforced by π–π stacking inter­actions. These latter contacts [*Cg*1· · ·*Cg*3 = 2.5382 (9) Å, *Cg*2· ··*Cg*2^ii^ = 3.5997 (9) Å, *Cg*2· · ·*Cg*3^iv^ = 3.6406 (10) Å and *Cg*3·· ·*Cg*2^v^ = 3.6406 (10) Å; symmetry codes: (ii) 1 − *x*, 1 − *y*, 1 − *z*; (iv) 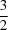 − *x*, 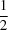 + *y*, 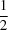 − *z*; (v) 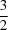 − *x*, −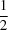 + *y*, 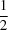 − *z*] also contribute to the stabilization of the crystal packing (Fig. 3 ▸).

## Database survey   

A search of the Cambridge Structural Database (Version 5.38, update February 2017; Groom *et al.*, 2016 ▸) revealed the structures of various organo­ammonium squarates (Georgopoulos *et al.*, 2005 ▸; Wang & Stucky, 1974 ▸; Kanters *et al.*, 1991 ▸; Kolev *et al.*, 2000 ▸; Karle *et al.*, 1996 ▸; Angelova *et al.*,1996 ▸). In the squarate anion form, the anions are generally linked to amines by N—H⋯O hydrogen bonds (Gilli *et al.*, 2001 ▸; Korkmaz *et al.*, 2011 ▸; Dega-Szafran *et al.*, 2012 ▸). Structures of 2-(amoniometh­yl) pyridinium, di­hydrogen squarate and squaric acid derivatives are also known (Korkmaz *et al.*, 2011 ▸; Korkmaz & Bulut, 2013 ▸, 2014 ▸). Often, the supra­molecular architectures of similar structures have been investigated together with their spectroscopic properties, including their potential non-linear optical (NLO) behaviour (Bosshard *et al.*, 1995 ▸; Kolev *et al.*, 2008 ▸). The literature also reveals that squarenes show photo-chemical, photo-conductive and NLO properties and that they can be used as electron acceptors in photo-sensitive devices (Korkmaz *et al.*, 2016 ▸).

## Synthesis and crystallization   

All chemical reagents were analytical grade commercial products. The solvent was purified by conventional methods. Squaric acid (H_2_Sq; 0,46 g, 4 mmol) and 2-(amino­meth­yl)pyridine (0,24 g; 2 mmol) were dissolved in water (25 cm^3^) to obtain a mixture in the molar ratio 2:1 and the solution was heated to 323 K in a temperature-controlled bath and stirred for one h. The reaction mixture was then slowly cooled to room temperature. The crystals formed were filtered and washed with 10 cm^3^ of methanol, and dried in air.

## Refinement   

Crystal data, data collection and structure refinement details are summarized in Table 2 ▸. All C-bound hydrogen atoms were included in calculated positions with C—H = 0.93 Å (aromatic) and 0.97 Å (methyl­ene) and allowed to ride, with *U*_iso_(H) = 1.2*U*_eq_(C). The NH_3_ group (N—H = 0.89 Å) was also allowed to ride in the refinement with *U*_iso_(H) = 1.5*U*_eq_(N). The O-bound H atoms and N1-bound H atom were located in a difference-Fourier map and refined with *U*_iso_(H) = 1.2*U*_eq_(O) and *U*_iso_(H) = 1.5*U*_eq_(N).

## Supplementary Material

Crystal structure: contains datablock(s) I. DOI: 10.1107/S2056989017004376/sj5524sup1.cif

Structure factors: contains datablock(s) I. DOI: 10.1107/S2056989017004376/sj5524Isup2.hkl

Supporting information file. DOI: 10.1107/S2056989017004376/sj5524Isup3.cml

CCDC reference: 1538918

Additional supporting information:  crystallographic information; 3D view; checkCIF report

## Figures and Tables

**Table 1:** Hydrogen-bond geometry (Å, °) *Cg*2 and *Cg*3 are the centroids of the C7–C10 and C11–C14 rings, respectively.

*D*—H⋯*A*	*D*—H	H⋯*A*	*D*⋯*A*	*D*—H⋯*A*
N1—H1*A*⋯O1	0.93 (2)	1.74 (2)	2.6598 (15)	166.7 (19)
N2—H2*B*⋯O2^i^	0.89	2.05	2.8565 (16)	150
N2—H2*B*⋯O5^i^	0.89	2.56	3.1613 (17)	126
N2—H2*C*⋯O6^i^	0.89	2.02	2.8765 (16)	161
N2—H2*A*⋯O8^ii^	0.89	1.90	2.7580 (15)	162
O4—H4*A*⋯O6^iii^	0.99 (2)	1.51 (2)	2.4993 (14)	175 (2)
O5—H5*A*⋯O3	1.00 (2)	1.46 (2)	2.4583 (14)	175 (2)
C7—O1⋯*Cg*2^ii^	1.25 (1)	3.38 (1)	3.3226 (14)	77 (1)
C7—O1⋯*Cg*3^iv^	1.25 (1)	3.39 (1)	3.3297 (14)	76 (1)
C13—O7⋯*Cg*2^v^	1.21 (1)	3.52 (1)	3.4188 (15)	75 (1)

Symmetry codes: (i) 
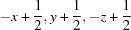
; (ii) 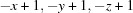; (iii) 
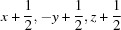
; (iv) 
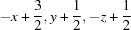
; (v) 
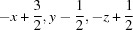
.

**Table 2:** Experimental details

Crystal data	
Chemical formula	C_6_H_10_N_2_^2+^·2C_4_HO_4_^−^
*M*_r_	336.26
Crystal system, space group	Monoclinic, *P*2_1_/*n*
Temperature (K)	296
*a*, *b*, *c* (Å)	7.4653 (7), 15.4548 (14), 12.2095 (12)
β (°)	90.073 (4)
*V* (Å^3^)	1408.7 (2)
*Z*	4
Radiation type	Mo *K*α
μ (mm^−1^)	0.13
Crystal size (mm)	0.17 × 0.13 × 0.11

Data collection	
Diffractometer	Bruker APEXII CCD
Absorption correction	Multi-scan (*SADABS*; Bruker, 2013 ▸)
*T*_min_, *T*_max_	0.672, 0.746
No. of measured, independent and observed [*I* > 2σ(*I*)] reflections	70681, 3496, 3090
*R*_int_	0.042
(sin θ/λ)_max_ (Å^−1^)	0.668

Refinement	
*R*[*F* ^2^ > 2σ(*F* ^2^)], *wR*(*F* ^2^), *S*	0.042, 0.118, 1.11
No. of reflections	3496
No. of parameters	227
H-atom treatment	H-atom parameters not refined
Δρ_max_, Δρ_min_ (e Å^−3^)	0.26, −0.28

Computer programs: *APEX2* and *SAINT* (Bruker, 2013 ▸), *SHELXT2014* (Sheldrick, 2008 ▸), *SHELXL2016* (Sheldrick, 2015 ▸), *ORTEP-3 for Windows* and *WinGX* (Farrugia, 2012 ▸) and *PLATON* (Spek, 2009 ▸).

## supplementary crystallographic information

### Crystal data

**Table:**

C_6_H_10_N_2_^2+^·2C_4_HO_4_^−^	*F*(000) = 696
*M**_r_* = 336.26	*D*_x_ = 1.586 Mg m^−^^3^
Monoclinic, *P*2_1_/*n*	Mo *K*α radiation, λ = 0.71073 Å
*a* = 7.4653 (7) Å	Cell parameters from 9896 reflections
*b* = 15.4548 (14) Å	θ = 3.0–28.3°
*c* = 12.2095 (12) Å	µ = 0.13 mm^−^^1^
β = 90.073 (4)°	*T* = 296 K
*V* = 1408.7 (2) Å^3^	Block, bronze
*Z* = 4	0.17 × 0.13 × 0.11 mm

### Data collection

**Table:**

Bruker APEXII CCD diffractometer	3090 reflections with *I* > 2σ(*I*)
φ and ω scans	*R*_int_ = 0.042
Absorption correction: multi-scan (SADABS; Bruker, 2013)	θ_max_ = 28.3°, θ_min_ = 3.0°
*T*_min_ = 0.672, *T*_max_ = 0.746	*h* = −9→9
70681 measured reflections	*k* = −20→20
3496 independent reflections	*l* = −16→16

### Refinement

**Table:**

Refinement on *F*^2^	0 restraints
Least-squares matrix: full	Hydrogen site location: mixed
*R*[*F*^2^ > 2σ(*F*^2^)] = 0.042	H-atom parameters not refined
*wR*(*F*^2^) = 0.118	*w* = 1/[σ^2^(*F*_o_^2^) + (0.0556*P*)^2^ + 0.5691*P*] where *P* = (*F*_o_^2^ + 2*F*_c_^2^)/3
*S* = 1.11	(Δ/σ)_max_ < 0.001
3496 reflections	Δρ_max_ = 0.26 e Å^−^^3^
227 parameters	Δρ_min_ = −0.28 e Å^−^^3^

### Special details

**Table:**

Geometry. All esds (except the esd in the dihedral angle between two l.s. planes) are estimated using the full covariance matrix. The cell esds are taken into account individually in the estimation of esds in distances, angles and torsion angles; correlations between esds in cell parameters are only used when they are defined by crystal symmetry. An approximate (isotropic) treatment of cell esds is used for estimating esds involving l.s. planes.

### Fractional atomic coordinates and isotropic or equivalent isotropic displacement parameters (Å^2^)

**Table:**

	*x*	*y*	*z*	*U*_iso_*/*U*_eq_	
C1	0.3472 (2)	0.59963 (10)	0.10814 (12)	0.0377 (3)	
H1	0.376471	0.541181	0.111071	0.045*	
C2	0.2856 (2)	0.63543 (13)	0.01196 (13)	0.0479 (4)	
H2	0.270245	0.601292	−0.050069	0.058*	
C3	0.2473 (2)	0.72223 (14)	0.00899 (14)	0.0507 (4)	
H3	0.210063	0.747750	−0.056155	0.061*	
C4	0.2640 (2)	0.77205 (11)	0.10321 (14)	0.0442 (4)	
H4	0.236500	0.830733	0.101700	0.053*	
C5	0.32152 (18)	0.73381 (8)	0.19877 (12)	0.0305 (3)	
C6	0.3378 (2)	0.78078 (10)	0.30627 (14)	0.0419 (4)	
H6A	0.424710	0.751293	0.352098	0.050*	
H6B	0.381247	0.839005	0.293104	0.050*	
C7	0.61186 (16)	0.53092 (8)	0.38741 (10)	0.0245 (2)	
C8	0.59463 (17)	0.44771 (8)	0.32689 (10)	0.0273 (3)	
C9	0.71303 (18)	0.40741 (8)	0.41129 (10)	0.0278 (3)	
C10	0.72538 (16)	0.48889 (8)	0.46610 (10)	0.0247 (2)	
C11	0.64141 (18)	0.16148 (8)	0.29601 (10)	0.0289 (3)	
C12	0.54027 (17)	0.10155 (8)	0.23404 (10)	0.0271 (3)	
C13	0.57553 (18)	0.03307 (9)	0.31604 (10)	0.0293 (3)	
C14	0.67917 (17)	0.09946 (8)	0.38172 (10)	0.0272 (3)	
N1	0.36494 (15)	0.64919 (7)	0.19743 (9)	0.0288 (2)	
N2	0.16375 (17)	0.78523 (8)	0.36470 (10)	0.0346 (3)	
H2A	0.177821	0.813962	0.427290	0.052*	
H2C	0.125260	0.731888	0.378838	0.052*	
H2B	0.083844	0.812501	0.322984	0.052*	
O1	0.54865 (14)	0.60498 (6)	0.37564 (8)	0.0328 (2)	
O2	0.51318 (15)	0.42458 (7)	0.24462 (8)	0.0387 (3)	
O3	0.77376 (16)	0.33276 (6)	0.42957 (9)	0.0409 (3)	
O4	0.80146 (14)	0.51691 (6)	0.55513 (8)	0.0331 (2)	
O5	0.68219 (19)	0.24159 (7)	0.27431 (9)	0.0457 (3)	
O6	0.45452 (15)	0.10825 (7)	0.14494 (8)	0.0380 (3)	
O7	0.54053 (18)	−0.04333 (7)	0.32469 (10)	0.0514 (3)	
O8	0.76167 (16)	0.09899 (7)	0.46951 (8)	0.0400 (3)	
H1A	0.419 (3)	0.6260 (13)	0.2598 (17)	0.048*	
H5A	0.725 (3)	0.2770 (14)	0.3380 (18)	0.060*	
H4A	0.868 (3)	0.4687 (15)	0.5894 (17)	0.060*	

### Atomic displacement parameters (Å^2^)

**Table:**

	*U*^11^	*U*^22^	*U*^33^	*U*^12^	*U*^13^	*U*^23^
C1	0.0412 (7)	0.0344 (7)	0.0375 (8)	0.0016 (6)	0.0005 (6)	−0.0072 (6)
C2	0.0440 (8)	0.0679 (11)	0.0320 (7)	−0.0028 (8)	−0.0042 (6)	−0.0111 (7)
C3	0.0442 (9)	0.0732 (12)	0.0346 (8)	0.0039 (8)	−0.0084 (6)	0.0176 (8)
C4	0.0458 (8)	0.0362 (8)	0.0505 (9)	0.0057 (6)	−0.0058 (7)	0.0159 (7)
C5	0.0293 (6)	0.0253 (6)	0.0370 (7)	−0.0015 (5)	−0.0047 (5)	0.0011 (5)
C6	0.0383 (7)	0.0333 (7)	0.0540 (9)	−0.0031 (6)	−0.0126 (7)	−0.0137 (6)
C7	0.0285 (6)	0.0245 (6)	0.0207 (5)	−0.0021 (4)	−0.0029 (4)	0.0028 (4)
C8	0.0327 (6)	0.0254 (6)	0.0237 (6)	−0.0010 (5)	−0.0045 (5)	0.0012 (5)
C9	0.0344 (6)	0.0230 (6)	0.0259 (6)	−0.0008 (5)	−0.0073 (5)	−0.0009 (4)
C10	0.0297 (6)	0.0222 (5)	0.0223 (5)	−0.0007 (4)	−0.0045 (4)	0.0009 (4)
C11	0.0364 (6)	0.0242 (6)	0.0261 (6)	0.0020 (5)	−0.0054 (5)	−0.0052 (5)
C12	0.0307 (6)	0.0257 (6)	0.0248 (6)	0.0040 (5)	−0.0059 (5)	−0.0052 (5)
C13	0.0322 (6)	0.0280 (6)	0.0276 (6)	−0.0013 (5)	−0.0061 (5)	−0.0020 (5)
C14	0.0297 (6)	0.0287 (6)	0.0234 (6)	0.0000 (5)	−0.0039 (5)	−0.0035 (5)
N1	0.0310 (5)	0.0265 (5)	0.0287 (5)	0.0017 (4)	−0.0043 (4)	0.0019 (4)
N2	0.0473 (7)	0.0276 (6)	0.0288 (6)	0.0021 (5)	−0.0120 (5)	−0.0032 (4)
O1	0.0431 (5)	0.0240 (4)	0.0312 (5)	0.0049 (4)	−0.0081 (4)	0.0028 (4)
O2	0.0505 (6)	0.0340 (5)	0.0315 (5)	−0.0006 (4)	−0.0189 (4)	−0.0030 (4)
O3	0.0594 (7)	0.0227 (5)	0.0405 (6)	0.0071 (4)	−0.0227 (5)	−0.0051 (4)
O4	0.0452 (5)	0.0257 (5)	0.0282 (5)	0.0014 (4)	−0.0150 (4)	−0.0028 (4)
O5	0.0797 (9)	0.0227 (5)	0.0346 (5)	−0.0061 (5)	−0.0174 (5)	−0.0021 (4)
O6	0.0520 (6)	0.0309 (5)	0.0310 (5)	0.0043 (4)	−0.0204 (4)	−0.0050 (4)
O7	0.0700 (8)	0.0309 (6)	0.0532 (7)	−0.0154 (5)	−0.0214 (6)	0.0067 (5)
O8	0.0525 (6)	0.0386 (6)	0.0290 (5)	−0.0023 (5)	−0.0171 (4)	−0.0034 (4)

### Geometric parameters (Å, º)

**Table:**

C1—N1	1.3388 (18)	C9—O3	1.2593 (16)
C1—C2	1.377 (2)	C9—C10	1.4291 (17)
C1—H1	0.9300	C10—O4	1.3000 (15)
C2—C3	1.372 (3)	C11—O5	1.3023 (17)
C2—H2	0.9300	C11—C12	1.4139 (17)
C3—C4	1.390 (3)	C11—C14	1.4465 (18)
C3—H3	0.9300	C12—O6	1.2658 (15)
C4—C5	1.376 (2)	C12—C13	1.4802 (18)
C4—H4	0.9300	C13—O7	1.2141 (17)
C5—N1	1.3475 (17)	C13—C14	1.5141 (17)
C5—C6	1.505 (2)	C14—O8	1.2356 (16)
C6—N2	1.485 (2)	N1—H1A	0.93 (2)
C6—H6A	0.9700	N2—H2A	0.8900
C6—H6B	0.9700	N2—H2C	0.8900
C7—O1	1.2462 (15)	N2—H2B	0.8900
C7—C10	1.4357 (16)	O3—H5A	1.46 (2)
C7—C8	1.4886 (17)	O4—H4A	0.99 (2)
C8—O2	1.2268 (15)	O5—H5A	1.00 (2)
C8—C9	1.4929 (17)		
			
N1—C1—C2	119.82 (15)	C10—C9—C8	89.62 (10)
N1—C1—H1	120.1	O4—C10—C9	135.48 (12)
C2—C1—H1	120.1	O4—C10—C7	131.74 (12)
C3—C2—C1	119.01 (15)	C9—C10—C7	92.73 (10)
C3—C2—H2	120.5	O5—C11—C12	129.70 (12)
C1—C2—H2	120.5	O5—C11—C14	137.03 (12)
C2—C3—C4	120.09 (15)	C12—C11—C14	93.24 (11)
C2—C3—H3	120.0	O6—C12—C11	132.49 (13)
C4—C3—H3	120.0	O6—C12—C13	136.82 (12)
C5—C4—C3	119.43 (15)	C11—C12—C13	90.68 (10)
C5—C4—H4	120.3	O7—C13—C12	135.73 (12)
C3—C4—H4	120.3	O7—C13—C14	136.29 (13)
N1—C5—C4	118.78 (14)	C12—C13—C14	87.94 (10)
N1—C5—C6	117.37 (12)	O8—C14—C11	136.71 (12)
C4—C5—C6	123.85 (14)	O8—C14—C13	135.18 (12)
N2—C6—C5	111.82 (11)	C11—C14—C13	88.10 (10)
N2—C6—H6A	109.3	C1—N1—C5	122.79 (12)
C5—C6—H6A	109.3	C1—N1—H1A	119.1 (12)
N2—C6—H6B	109.3	C5—N1—H1A	117.8 (12)
C5—C6—H6B	109.3	C6—N2—H2A	109.5
H6A—C6—H6B	107.9	C6—N2—H2C	109.5
O1—C7—C10	135.70 (12)	H2A—N2—H2C	109.5
O1—C7—C8	134.73 (11)	C6—N2—H2B	109.5
C10—C7—C8	89.54 (10)	H2A—N2—H2B	109.5
O2—C8—C7	134.48 (12)	H2C—N2—H2B	109.5
O2—C8—C9	137.39 (12)	C9—O3—H5A	108.5 (9)
C7—C8—C9	88.12 (9)	C10—O4—H4A	108.8 (12)
O3—C9—C10	134.53 (12)	C11—O5—H5A	115.9 (12)
O3—C9—C8	135.83 (12)		
			
N1—C1—C2—C3	1.5 (2)	O1—C7—C10—C9	177.87 (16)
C1—C2—C3—C4	−2.5 (3)	C8—C7—C10—C9	−0.11 (10)
C2—C3—C4—C5	0.9 (3)	O5—C11—C12—O6	−4.0 (3)
C3—C4—C5—N1	1.8 (2)	C14—C11—C12—O6	177.80 (15)
C3—C4—C5—C6	−177.64 (15)	O5—C11—C12—C13	176.91 (15)
N1—C5—C6—N2	−96.31 (15)	C14—C11—C12—C13	−1.33 (11)
C4—C5—C6—N2	83.15 (19)	O6—C12—C13—O7	4.0 (3)
O1—C7—C8—O2	0.6 (3)	C11—C12—C13—O7	−176.90 (19)
C10—C7—C8—O2	178.65 (16)	O6—C12—C13—C14	−177.79 (17)
O1—C7—C8—C9	−177.91 (15)	C11—C12—C13—C14	1.27 (10)
C10—C7—C8—C9	0.10 (10)	O5—C11—C14—O8	2.2 (3)
O2—C8—C9—O3	−0.3 (3)	C12—C11—C14—O8	−179.74 (17)
C7—C8—C9—O3	178.15 (17)	O5—C11—C14—C13	−176.71 (18)
O2—C8—C9—C10	−178.57 (17)	C12—C11—C14—C13	1.30 (10)
C7—C8—C9—C10	−0.11 (10)	O7—C13—C14—O8	−2.1 (3)
O3—C9—C10—O4	−0.8 (3)	C12—C13—C14—O8	179.77 (16)
C8—C9—C10—O4	177.53 (16)	O7—C13—C14—C11	176.91 (19)
O3—C9—C10—C7	−178.19 (17)	C12—C13—C14—C11	−1.24 (10)
C8—C9—C10—C7	0.11 (10)	C2—C1—N1—C5	1.3 (2)
O1—C7—C10—O4	0.3 (3)	C4—C5—N1—C1	−3.0 (2)
C8—C7—C10—O4	−177.69 (15)	C6—C5—N1—C1	176.53 (13)

### Hydrogen-bond geometry (Å, º)

Cg2 and Cg3 are the centroids of the C7–C10 and C11–C14 rings, respectively.

**Table:**

*D*—H···*A*	*D*—H	H···*A*	*D*···*A*	*D*—H···*A*
N1—H1*A*···O1	0.93 (2)	1.74 (2)	2.6598 (15)	166.7 (19)
N2—H2*B*···O2^i^	0.89	2.05	2.8565 (16)	150
N2—H2*B*···O5^i^	0.89	2.56	3.1613 (17)	126
N2—H2*C*···O6^i^	0.89	2.02	2.8765 (16)	161
N2—H2*A*···O8^ii^	0.89	1.90	2.7580 (15)	162
O4—H4*A*···O6^iii^	0.99 (2)	1.51 (2)	2.4993 (14)	175 (2)
O5—H5*A*···O3	1.00 (2)	1.46 (2)	2.4583 (14)	175 (2)
C7—O1···*Cg*2^ii^	1.25 (1)	3.38 (1)	3.3226 (14)	77 (1)
C7—O1···*Cg*3^iv^	1.25 (1)	3.39 (1)	3.3297 (14)	76 (1)
C13—O7···*Cg*2^v^	1.21 (1)	3.52 (1)	3.4188 (15)	75 (1)

Symmetry codes: (i) −*x*+1/2, *y*+1/2, −*z*+1/2; (ii) −*x*+1, −*y*+1, −*z*+1; (iii) *x*+1/2, −*y*+1/2, *z*+1/2; (iv) −*x*+3/2, *y*+1/2, −*z*+1/2; (v) −*x*+3/2, *y*−1/2, −*z*+1/2.
